# Modern Medico-Psychology and Psychiatry

**Published:** 1894-07-07

**Authors:** T. S. Clouston

**Affiliations:** Lecturer on Mental Diseases, Edinburgh University, and Physician Superintendent Royal Asylum, Morningside


### Modern Medico-Psychology and Psychiatry.

By T. S. Clouston, M.D., Lecturer on Mental Diseases, Edinburgh University, and Physician Superintendent Royal Asylum, Morningside.

### IX.—FORMS AND CLASSIFICATION OF MENTAL DISEASE—(*continued*).

As to the details of medical and dietetic treatment this is not the place to enter fully, but quinine, iron, strychnine, strophanthus, and digitalis, the mineral acids, the phosphates, the hypophosphites, the vegetable bitters, some of the more tonic and gentle laxative mineral waters, cholagogues, and diaphoretics are all most useful in different cases. Of course the diathesis of the patient must be kept in view to guide us in using drugs, and exhaustive inquiry into what organ or function is specially disturbed must always be made to guide us in putting it right. A melancholic may be so because his brain is being poisoned by morbid products from disordered working of the stomach, liver, bowels, kidneys, or skin, circulating in his blood. We have much scientific confirmation from physiological experiments as to how some of the medical agents I have mentioned act. Roy and Sherrington, to take an example, have shown that the mineral acids and strychnine visibly stimulate the vaso-motor apparatus in the brain and determine more blood to its nervous elements.

The question of how the insomnia of melancholia is to be got over is one of the most difficult of all the problems the psychiatrist has to face. Are drug hypnotics and brain sedatives to be used or not? Beyond any doubt they have their uses, and it is certain also they have their great dangers and disadvantages, and may be abused. A mild alcoholic stimulant, like ale or porter or whisky and water, will often act as an unobjectionable and most efficient sleep producer. Paraldehyde is chemically analogous to an alcohol and its sleep is often refreshing and restorative almost like "tired nature's sweet restorer." Chloral and the bromides and cannabis and even opium come in useful sometimes. Chloralamide, urethane and sulphonal are often useful. But the word "beware" should be written on every draught bottle containing any of these hypnotics. One hour's perfectly normal sleep is usually worth several hours' drug sleep. Massage and the judicious use of a tumbler of hot water at night, hot and cold baths, or of hot bottles, packs and cold sponging, with subsequent friction of the legs at bed-time are often useful. The uses of all these agents are founded on physiological knowledge and medical experience. During sleep the blood largely leaves the brain and it becomes anæmic, therefore the agents that dilate the blood-vessels elsewhere take away the blood from the brain and so may induce sleep.

As to diet, the principles of feeding a melancholic are perfectly simple, its details are often very complicated and difficult. Get his body and his brain well nourished by simple, digestible, tempting food. To do this you have often to consult his tastes, and humour his whims. Codliver oil, maltine, pancreatic emulsion, milk, eggs, cream, fruits, butter, saccharine materials, farinaceous diet, the lighter kinds of animal food, experience has found to be curative to melancholia, because they nourish the atonic and wasted brain cells. I have long preached the gospel of fatness to my melancholics. To fatten them is to cure them. Mrs. Carlyle well exclaimed "Oh thank God for the precious layer of impassivity which that stone weight of fat has put over my nerves." Burton said long ago, "Make a melancholy man fat, as Rhasis saith, and thou hast finished the cure."

There are many varieties of melancholia, some of which do not correspond to the true and common type I have described. There are the overfed, fat, and self-indulgent melancholics; there are those whose mental symptoms seem to be summed by the words egotism and selfishness. Cures are sometimes effected far otherwise than by rest, tonics, and feeding; violent purgation, severe bleedings, fevers, bodily illnesses, mental shocks, loss of money and friends have all been known effectually to rouse and cure certain kinds of melancholic patients. But these are the exceptions that prove the rule.

As to its heredity, beyond any doubt melancholia commonly arises out of an innate oversensitive, unstable condition of brain derived from ancestry. The man commonly takes it from his mother or her family, the woman from the father. But what disease is not hereditary more or less? A bad heredity does not mean incurability, but often the reverse; a brain that is easily upset is often easily put to rights.

